# A New Version of the Beuchet Chair Illusion

**DOI:** 10.1177/2041669516679168

**Published:** 2016-11-22

**Authors:** Richard Wiseman

**Affiliations:** University of Hertfordshire, UK

**Keywords:** illusion, perception, size, vision

## Abstract

The Beuchet Chair is a powerful and highly popular optical illusion. The illusion involves two reasonably large pieces of apparatus: an oversized chair seat and four normal-sized chair legs. When properly arranged and viewed from a precise location, a person standing on the seat appears to be much smaller than they actually are. Although compelling, the illusion is relatively challenging and expensive to construct, requires a large amount of space to stage, and is not especially portable. Here, I outline a new version of the illusion that just involves a small piece of cardboard, a cloth, and a tripod. This new version costs almost nothing to create, is highly portable, and requires far less space than the original.

Educational institutions and visitor attractions across the world regularly use the Beuchet Chair illusion to entertain and educate. Created by French psychologist Jean Beuchet (1963), the illusion involves two pieces of apparatus: an oversized chair seat and four normal-sized chair legs. The four chair legs are arranged in an upright position a few meters from an observer, and the seat is placed on the ground a few meters beyond the legs. When observed with monocular vision, the seat and legs visually fuse together to create what appears to be a normal chair. As a result, the observer perceives the chair seat to be much closer than it actually is, and so anyone standing or sitting on the chair seat appears to be much smaller than they actually are.

Those wishing to stage this powerful and compelling illusion currently face several practical challenges. First, constructing the two pieces of apparatus is far from easy as it involves building an oversized seat and cutting the tops of each of the chair legs at a precise angle. Second, the resulting apparatus is not especially portable and takes up a considerable amount of storage space. Finally, the illusion can only be staged in a relatively large room because of the required distances between the observer, legs, and seat.

I recently devised a very different way of creating the illusion that overcomes all of these difficulties. This new version of the chair illusion simply involves a 20-cm cardboard template of a chair, a 2.5 m × 1.5 m piece of brightly coloured cloth, and a 60-cm high tripod. The *front* leg of the template is attached to the top of the tripod (see Figure 1), and the tripod placed about 25 cm from the observer. The cloth is then placed on the floor a few meters back from the tripod, and folded to ensure that it forms the chair seat when viewed from the desired position (see Figure 2). The resulting illusion is identical to the Beuchet Chair – namely, anyone standing on the cloth will appear to be much smaller than they actually are (see Figure 3). An alternative arrangement involves attaching the template to a piece of clear plastic, and taking the photograph through the plastic. Although this arrangement is possible (Wiseman, 2016a), it can be problematic to control the reflections on the plastic sheet.

**Figure 1.**
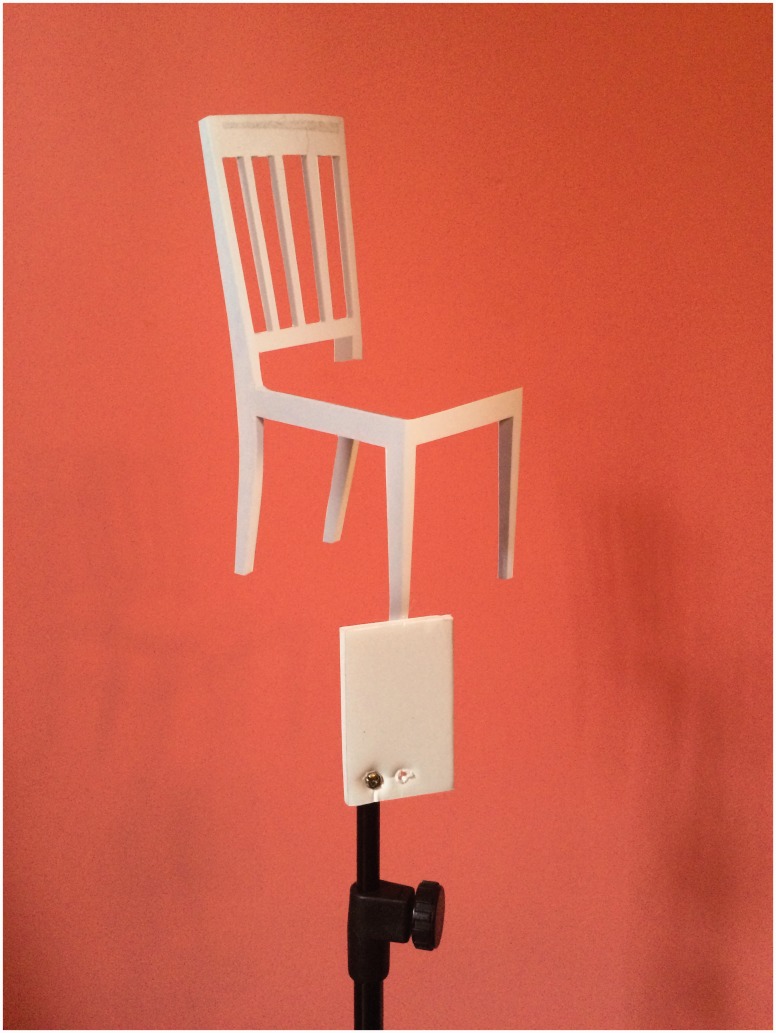
The chair template attached to the tripod.

**Figure 2.**
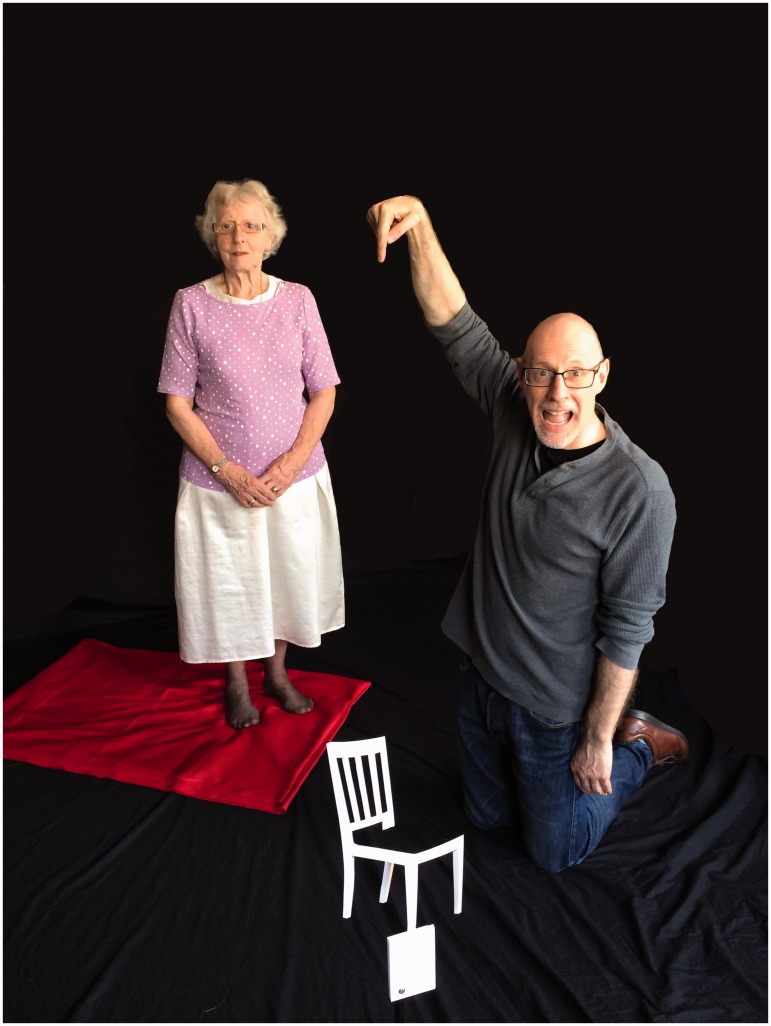
The arrangement for the Wiseman Chair.

**Figure 3.**
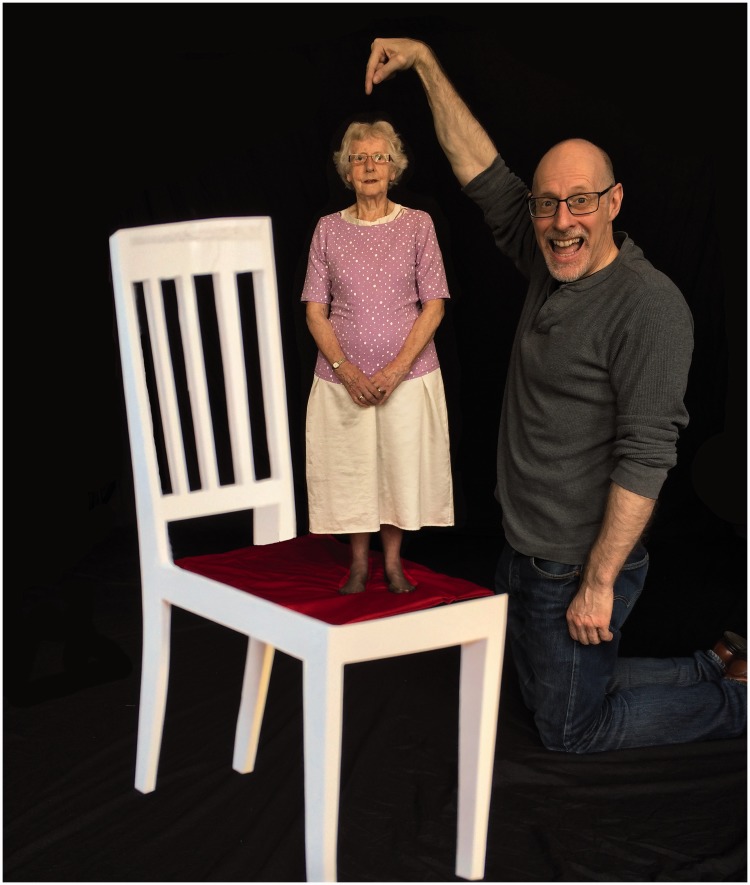
The Wiseman Chair illusion.

The method of creating the illusion is somewhat different to the one used in the original Beuchet Chair and the template proved challenging to design. First, all of the items involved in the original illusion are relatively large, and so a camera does not struggle to focus on the various elements. However, in the new version, the chair is much smaller and very close to the camera, which could result in focusing issues. Because of this, it was necessary to create a template that was large enough to create the required illusion, but not so large that it completely dominated the field of view and therefore caused the camera to focus solely on the chair. Second, unlike the Beuchet Chair, the person who appears to be on the chair cannot seem to stand in front of the chair back. Because of this, the seat of the chair had to be widened to allow them to stand to the right of the chair back. Initial attempts resulted in a chair seat that looked unnaturally wide, but it eventually proved possible to create a design that does not arouse suspicion. Third, whereas the legs of the Beuchet Chair simply rest on the floor, the template has to be supported by the tripod. This was made possible by extending the front leg of the chair and again involved creating a design in which an extended leg did not appear suspicious.

This new version of the Beuchet chair costs very little to make, is extremely easy to construct and transport, takes up almost no storage space, and can be staged in a much smaller room than the traditional Beuchet chair. I briefly described the illusion in a recent blog post (Wiseman, 2016b) and was contacted by several researchers wishing to use it. I am happy to provide the template to those wishing to stage the illusion (see supplementary materials), and hope that this simplified version of Beuchet’s wonderful idea will result in many more people appearing to be much smaller than they actually are.

## Supplementary Material

Supplementary material

## Supplemental Material

The supplemental material is available at: http://IPE/sagepub.com/supplemental

## References

[bibr1-2041669516679168] Beuchet J. (1963). *La Chaise ŕductrice: Etude d’une illusion de grandeur et de distance* [The Reducing Chair: A study in the illusion of size and distance]. Paris: Service du Film de Recherche Scientifique. Retrieved from https://www.canal-u.tv/video/cerimes/la_chaise_reductrice_etude_d_une_illusion_de_grandeur_et_de_distance.9267.

[bibr2-2041669516679168] Wiseman, R. (2016a). Amazing Miniature Bear! Retrieved from https://www.youtube.com/watch?v=UwmaEocw1oY.

[bibr3-2041669516679168] Wiseman, R. (2016b). A new Beuchet chair illusion [Web blog post]. Retrieved from https://richardwiseman.wordpress.com/2016/08/07/a-new-beuchet-chair-illusion/. 10.1177/2041669516679168 PMC5131737 27928495

