# Supplementary figures and images for: A New Version of the Beuchet Chair Illusion
Source: Iperception. 2016 Nov 22;7(6):2041669516679168. doi: 10.1177/2041669516679168 (PMC5131737; doi:10.1177/2041669516679168)

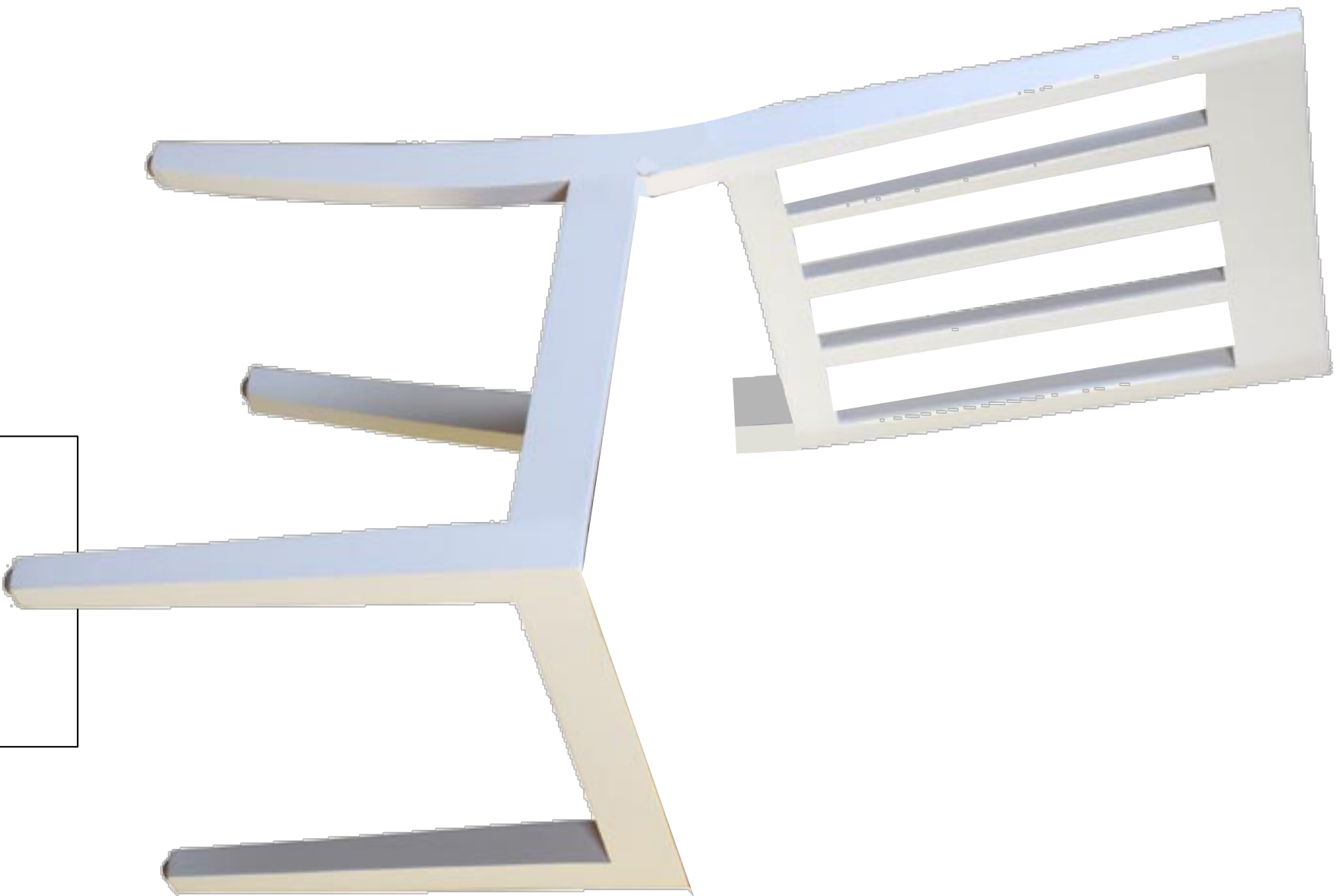

Leave this  
section attached  
to chair

Supplement: Supplementary material [file wisemanchair.pdf]
